# *Cannabis*-Based Oral Formulations for Medical Purposes: Preparation, Quality and Stability

**DOI:** 10.3390/ph14020171

**Published:** 2021-02-22

**Authors:** Francesca Baratta, Marco Simiele, Irene Pignata, Lorenzo Ravetto Enri, Antonio D’Avolio, Riccardo Torta, Anna De Luca, Massimo Collino, Paola Brusa

**Affiliations:** 1 Department of Drug Science and Technology, University of Turin, Via Pietro Giuria 9, 10125 Turin, Italy; irene.pignata@unito.it (I.P.); lorenzo.ravettoenri@unito.it (L.R.E.); massimo.collino@unito.it (M.C.); paola.brusa@unito.it (P.B.); 2 Laboratory of Clinical Pharmacology and Pharmacogenetics, Department of Medical Sciences, University of Turin, ASL “Città Di Torino”, Amedeo di Savoia Hospital, Corso Svizzera, 164, 10149 Turin, Italy; marco.simiele@coqualab.it (M.S.); antonio.davolio@unito.it (A.D.); 3 Academic Spin off CoQua Lab s.r.l, Corso Svizzera, 164, 10149 Turin, Italy; 4 Department of Neurosciences “Rita Levi Montalcini”, University of Turin, Via Cherasco 15, 10126 Turin, Italy; riccardo.torta@unito.it; 5 University Hospital “Citta della Salute e della Scienza di Torino”, Corso Bramante 88, 10126 Turin, Italy; adeluca@cittadellasalute.to.it

**Keywords:** medical *Cannabis*, *Cannabis* oil, THC, CBD, standard procedures, stability

## Abstract

Current legislation in Italy provides that medical *Cannabis* may be administered orally or by inhalation. One of the fundamental criteria for the administration of oral formulations is that they deliver a known consistent quantity of the active ingredients to ensure uniform therapies leading to the optimisation of the risks/benefits. In 2018, our group developed an improved *Cannabis* oil extraction technique. The objective of the present work was to carry out a stability study for the oil extracts obtained by this method. Furthermore, in order to facilitate the consumption of the prescribed medical *Cannabis* therapy by patients, a standard procedure was defined for the preparation of a single-dose preparation for oral use (hard capsules) containing the oil extract; thereafter, the quality and stability were evaluated. The hard capsules loaded with the oil extract were analysed and found to be uniform in content. The encapsulation process did not alter the quantity of the active molecule present in the oil. The stability tests yielded excellent results. Since the capsule dosage form is easily transported and administered, has pleasant organoleptic properties and is stable at room temperature for extended periods of time, this would facilitate the adherence to therapy by patients in treatment.

## 1. Introduction

Before the last century, when it became illegal in most Countries, *Cannabis* was widely used in medicine. The reason for the restriction in its availability as a therapeutic agent was its growing notoriety as a psychotropic agent and its consequent abuse [1,2]. In recent years, however, there has been a resurgence in support for the legalisation of cannabinoids for medical use as a result of media attention as well as expectations of their efficacy, albeit, this is not always supported by scientific evidence [3,4,5].

The phytocomplex of the *Cannabis* plant contains over 500 different molecules, of which approximately a hundred belong to the cannabinoid chemical class; among these, small differences in molecular structure may induce widely different effects [6]. The molecules of greatest pharmacological interest from the point of view of their effects are the decarboxylated forms of delta-9-tetrahydrocannabinol (THC) and cannabidiol (CBD) as these are easily absorbed in the intestine [7]. Hence, the determination of the quantities of these compounds present in medications to be administered to patients is a fundamental prerequisite.

In the last few years, reforms in Italy have opened the door to the use of medical *Cannabis* in tightly regulated cases. Consequently, *Cannabis* is now available for this purpose. In Italy medical *Cannabis* is produced by the Stabilimento Chimico Farmaceutico Militare (Pharmaceutical Chemical Military Facility) in Florence.

Since 2016, a variety of *Cannabis*, FM2, has been available. This is supplied as dried, ground *Cannabis* inflorescences containing delta-9-tetrahydrocannabinol in quantities ranging from 5% to 8% and cannabidiol in percentages from 7.5% to 12%. Since 2018, another variety of *Cannabis*, FM1, has also been made available; this contains delta-9-tetrahydrocannabinol in quantities from 13% to 20% while the cannabidiol content is lower than 1%. Note that the percentages reported refer to the “total” content: that is, the sum of the molecule in both acid form (delta-9-tetrahydrocannabinolic acid—THCA-and cannabidiolic acid—CBDA) and decarboxylated form (delta-9-tetrahydrocannabinol—THC and cannabidiol—CBD) [8,9,10].

Currently in Italy, the law states that medical *Cannabis* may be administered orally or by inhalation. The administration by inhaler is to be considered the second-choice option and must only be selected when oral administration does not produce the desired pharmacological effects or when the physician considers it opportune [9,11].

Concerning oral administration, in accordance with Minister of Health directives, decoctions represent the first-choice pharmaceutical form. The decoction must be prepared in compliance with the official procedure reported in “Recommendations for doctors prescribing FM2 *Cannabis* inflorescence derivatives” [11]. In a previous study, our research group demonstrated that the prescription of decoction-based formulations, considering the low yields of THC and CBD and, consequently, the high volume that a patient would have to consume as well as the high costs of processing the raw material to obtain the required quantity of active molecule, should not be recommended [12].

As well as the decoction for oral administration, the legislation in effect in Italy specifies that medical *Cannabis* may also be administered as an oil extract (hereafter, oil) on condition that this has been previously titrated for the active molecule using the proper instrumentation as set out by the current regulations (gas or liquid chromatography coupled with mass spectroscopy) [9,11]. The administration of formulations containing known quantities of active molecule is essential to ensure the uniformity of therapies leading to the consequent optimisation of the risks/benefits. On this point, it is important to note that although a number of preparation methods have been reported in scientific literature [13,14,15,16], the situation for oils, in particular, was that an exhaustive comparative study was lacking which investigated the technical aspects of preparation procedures for *Cannabis*-based formulations for medical purposes.

In light of this, in 2018, our research group developed a novel preparation method (denominated β-4) that allowed us to obtain a significantly higher amount of THC and CBD than those for water extraction (decoction) or an oil extraction using the previously known methods most widely used in Italy [12].

Having optimised the extraction procedure, the objective of the present study was to conduct stability studies on the oils obtained through the β-4 method. In addition, considering that oils have received considerable attention due to their easier dose management during the treatment period, but their organoleptic characteristics are particularly unpleasant, in order to facilitate the consumption of the prescribed medical *Cannabis* therapy by a patient in treatment, a standard procedure was defined for the preparation of a single-dose preparation for oral use (hard capsules) using the oil-based formulation. Both the oil and the capsules were then evaluated for quality and stability. Furthermore, the preparation of a pharmaceutical form that masks the organoleptic characteristics of the oil has the advantage of allowing the establishment of a control/placebo group in a clinical trial.

## 2. Results

### 2.1. Capsules Preparation

Using the oils obtained with the β-4 procedure, rigid capsules were prepared.

The optimal order in which the components should be added was experimentally evaluated. The most efficacious method was adding the olive oil first and then, the silica. Indeed, when we attempted to dispense oil into a capsule already containing a layer of silica, the presence of the excipient made the operation extremely difficult in that each drop of oil disturbed the powder and caused a small puff of the powder to drift out of the capsule given its fine composition. It was more practical to add the oil first; cover it with a layer of silica and then, mix the two by inverting 180° the capsule filling machine to accelerate the mixing process.

### 2.2. Capsules’ Quality

The capsules, prepared with oils obtained with the β-4 method employing the technique described in Section 4.3, analysed employing the method described in Section 4.4, were uniform in mass and content and complied with the directives set out in the European Pharmacopeia [17] In particular, the weight of the individual dosage units and the amount of the lipophilic liquid phase present in the single capsules varied by less than 10% from the average value.

The titration of the active molecules for the oil extracted from the capsules varied by less than 10% from the expected result. Table 1 reports the titrated quantity of active molecules in the oil extracted from capsules prepared using two different volumes of oil (156 µL/cps and 312 µL/cps) deriving from the β-4 extraction procedure. As the results show, the quantity of oil extracted from the capsule is consistent with the expected result and independent of the quantity of oil dispensed into the capsule.

The encapsulation process, and, hence, the contact with the silica and the capsule gelatin, did not alter the quantity of the tested active molecules present in the oil independently of the quantity used to fill the capsule. Furthermore, no alteration of the envelope occurred: the use of different volumes of oil had also the purpose of evaluating whether alteration of the gelatin envelope occurred as the quantity of oil increased.

### 2.3. Stability Tests

The stability tests conducted employing the method described in Section 4.5 both on the oils obtained from the β-4 procedure and the capsules containing the same oil, yielded excellent results: the variation in the content of active molecules was less than 10% both after refrigerated storage for 180 days and after storage at room temperature for 180 days. This result was valid for both the oils and the oil in capsules. The maximum variation for oils was 9.29%, for capsules was 9.10%.

**Figure 1:**
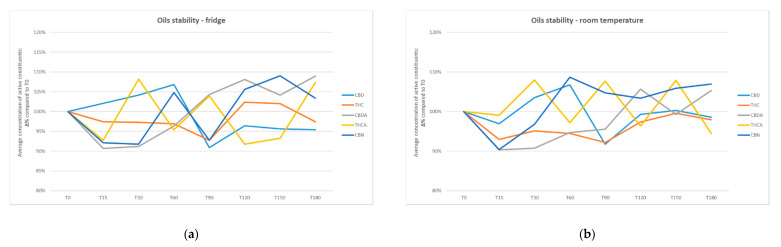
Oils stability: average concentration of active constituents compared to T0. (**a**) Oils stability in fridge; (**b**) oils stability at room temperature. T0: initial conditions; T 15: 15 days; T 30: 30 days; T 60: 60 days; T 90: 90 days; T 120: 120 days; T 150: 150 days; T 180: 180 days. Standard deviation for oils has been detailed in Table 2.

**Figure 2:**
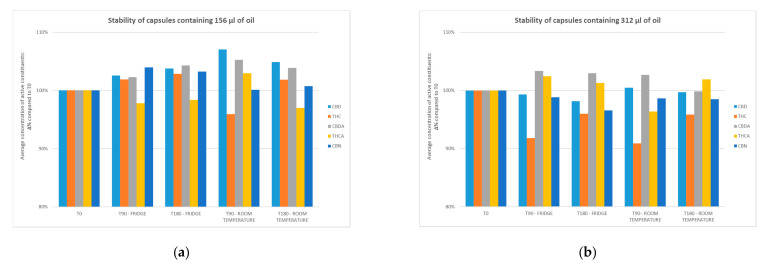
Capsules stability: average concentration of active constituents compared to T0. (**a**) Stability of capsules containing 156 µL of oil in fridge and at room temperature; (**b**) Stability of capsules containing 312 µL of oil in fridge and at room temperature. T0: initial conditions; T 90: 90 days; T 180: 180 days. Standard deviation for oils has been detailed in Table 3.

Table 2 and Table 3 respectively report the results of the analysis of three 100 mL batches of oil prepared using the β-4 procedure and the capsules filled with the same oils. Figure 1 and Figure 2 show the active molecules concentrations in oils and capsules during 180 days: the variations were always less than 10.

## 3. Discussion

In 2018 our research group developed a novel preparation method (denominated β-4) and the results obtained using this procedure were compared with the three established techniques most widely used in Italy [12]. In detail, two of these techniques instruct that the *Cannabis* be, first, ground and then, mixed with olive oil. The resulting mixture is heated (for two hours in a water bath at boiling point for one method, and for two hours at 110 °C for the other) and then, filtered to obtain the oil extract. The third method, instead, directs that the *Cannabis* is chopped and then, pre-heated at 115 °C for 40 min. Subsequently, the *Cannabis* is mixed with olive oil and further ground with a turbo-emulsifier for three minutes. The mix of *Cannabis* and olive oil is then heated in a water bath at boiling point for 40 min, before being filtered and added with butylhydroxytoluene (BHT) 0.02%. The three examined methods specify that the weight (mg) to volume (mL) ratio between plant material and solvent is 100:1. Using type FM2 *Cannabis*, the most effective of the three established methods described above yields oil with a maximum concentration of THC equal to 0.37% ± 0.08% (3.38 mg/mL) and that of CBD is equal to 0.70% ± 0.19% (6.40 mg/mL) [12,13,14,15,16]. The β-4 method revealed itself to be the most effective: using a quantity of vegetable material twice that of the other methods (weight/volume ratio of plant material to solvent of 200:1), led to an average concentration of active molecules in the decarboxylated form more than double of the other methods: 8.04 mg/mL for THC and 13.05 mg/mL of CBD. These values represent significantly higher returns than those for water extraction (decoction) or an oil extraction using the previously known methods [12].

Considering the good results, we performed stability studies on the oils obtained through the β-4 method. In contrast to previous studies on oil preparation [14,18,19,20,21], the β-4 oils were stable up to 180 days not only if stored in the refrigerator, but also if stored at room temperature, regarding both the two major components -THC and CBD- and the others have been tested.

The stability has been the same for the capsules fitted with different amounts of the β-4 oils: the results revealed that the encapsulation process has no effect on the oils.

We did not performed tests and there is no evidence in the literature describing the losses of the phytocomplex components during the various stages of preparation [18]. The deepening of the losses occurred during the different preparation phases, which until now has been presented only in terms of concentrations of active compounds obtained in final oils, need to be investigated, in particular focused on decarboxylation.

The choice of the hard capsule as the dosage form for the oral route was tied to the fact that this is a relatively easy form to prepare and, in addition, the equipment required for this process is readily available in the majority of Italian hospital and community pharmacies.

The optimal encapsulation procedure was selected after a variety of tests. In the initial tests, the oil in its pure form without excipient was used to fill the capsule as it was presumed that this would not affect the capsule casing. However, while the casings appeared to be intact, on closer examination, the lack of an excipient to provide internal support caused it to be particularly fragile. Furthermore, the oil could leak from around the joint between the two parts of the capsule. Hence, it was decided to use an excipient such as silica able to gel the oil thus preventing the oil from leaking and solidifying the contents of the capsule.

Successively, the optimal order in which the components should be added was evaluated and a variety of preparation tests were conducted. These allowed us to understand that the most efficacious method was adding the olive oil first and then, the silica.

The development of dosage forms for oral use based on oil formulations aims to facilitate the consumption of the prescribed therapy by the patient in treatment with medical *Cannabis*. The encapsulation process offers numerous advantages: firstly, it masks the organoleptic properties of the formulation and this favours adherence to therapy by the patient. The oils in particular have particularly unpleasant organoleptic properties, which may have negative consequences for proper adherence. In addition, encapsulation makes handling and transportation easier; furthermore, considering the fact that storage at low temperature is not necessary for at least six months, the management of the prescribed therapy is certainly simplified for the patient even in terms of domestic storage of the dosage units.

The preparation of a single-dose unit to be administered orally, such as that developed in the course of the present study, allows the administration of a placebo in a clinical trial; this is an essential step considering the fact that, to date, the efficacy of medical *Cannabis* has not been demonstrated definitively by scientific literature and requires further study. One of the particular organoleptic properties of *Cannabis*-based formulations is the odour, which is intense and unmistakable; therefore, a subject or the experimenter would easily realize in which group the subject has been included. This would nullify the randomness of subjects thus rendering the entire experiment void.

## 4. Materials and Methods

### 4.1. Active Compounds

All of the galenic preparations described below were based on flowering tops from type FM2 *Cannabis* purchased from the Pharmaceutical Chemicals Military Facility in Florence. The titrated concentrations of active compounds in the unprocessed material (year 2017), evaluated as described in Section 4.4, were 0.40% ± 0.02% for THC, 5.74% ± 0.18% for THCA, 0.29% ± 0.03% for CBD and 8.70% ± 0.17% for CBDA. Consequently, the total THC, calculated by the formula %THC tot = %THC + (0.877 × %THCA), was 5.43% ± 0.15% and the total CBD content, calculated by the formula %CBD tot = %CBD + (0.877 × %CBDA), was 7.92% ± 0.18%. The formulae adjusted for the differing molecular weight of the cannabinoid and carboxylic conjugative components of each cannabinoid [19]. At the end of the experimentation period (year 2018), the titrated quantities of the active constituents in the plant material were reassessed and were 2.54% ± 0.33% for THC, 2.97% ± 0,41% for THCA, 1.71% ± 0.26% for CBD, 6.29% ± 0.72% for CBDA. It follows that the total THC was equal to 5.14% ± 0.69% and total CBD was 7.23% ± 0.95%. 

The method β-4 allows to obtain oils whose average concentration of active molecules in the decarboxylated form is 8.04 mg/mL for THC and 13.05 mg/mL of CBD [12].

### 4.2. Materials for the Galenic Preparation and Reagents for Quantitative Analysis

The other materials used for producing the galenic preparation described below (olive oil, distilled water, micronized silica anhydride, type 0 rigid capsules), were purchased from a pharmaceutical supplies company (Farmalabor s.r.l, Canosa di Puglia, Bari, Italy) and complied with the relevant monograph of the European Pharmacopoeia (Eur.Ph). The preparation of the capsules was performed using a 100-hole manual capsule filling machine (Farmalabor, Optima Aluminium^®^) and a precision pipette (Gilson, Microman^®^).

Regarding quantitative analysis, olive oil (pharmaceutical grade), CBD, cannabinol (CBN), CBDA, cannabidiol-d3 (CBD-d3), THC, (-)-delta9-tetrahydrocannabinol-d3 (THC-d3) and isopropanol LC-MS grade were purchased from Sigma–Aldrich (Milan, Italy). THCA was purchased from LGC (Milan, Italy). Acetonitrile LC-MS grade was purchased from VWR (Milan, Italy). HPLC grade water was produced with Elix-coupled with Synergy-UV water purification system (Merck Millipore, Milan, Italy). 

### 4.3. Oils and Capsules Preparation

For the preparation of the *Cannabis* oil, a precise quantity of FM2 *Cannabis* inflorescences was immersed in a precise quantity of extra-virgin olive oil in a weight to volume ratio of 200:1 (mg/mL). The oil containing the inflorescences was then placed in a water bath at boiling point with stirrer for 60 min. Subsequently, the oil was filtered using cotton gauze or hydrophilic cotton gauze in a manual press. Before the oil extraction phase, the *Cannabis* flowers had been ground for 60 s to produce a uniformly sized batch; spread in a thin layer (5 mm max; optimal thickness 1–2 mm) and placed in an oven at 140 °C for 30 min. The temperature applied was based on the fact that this is close to the evaporation point of THC (145 °C) [22]. The volume of the batches prepared was between 5 and 100 mL [12].

Using the oils obtained with the described procedure, rigid capsules were prepared. In detail, after opening the casing using a manual capsule filler, the oil was dispensed into the capsules in quantities from 0.1 mL to 0.4 mL using a precision pipette. The remaining space in each capsule was filled with micronized anhydrous silica in quantities ranging from 5 mg to 20 mg. Thereafter, the capsules were closed. The capsules were not removed from the capsule filling machine, but it was inverted 180° so that the oil would mix with the silica. The capsules were allowed to rest in this position for 12 h: after that, they were removed from the capsule filling machine.

### 4.4. Analytical Method

Chromatographic analysis [23] was performed by Acquity^®^ UPLC system coupled with a TQD mass spectrometer (Waters, Milan, Italy). The chromatographic separation was carried out using an Acquity UPLC HSS T3 column (2.1 × 30 mm, 1.8 µm) (Waters, Milan, Italy) at a constant 30 °C. The chromatographic separation was obtained by a gradient of mobile phases A (acetonitrile and water in a ratio of 70:30 + 0.05% Formic acid) and B (isopropanol and acetonitrile in a ratio of 80:30 + 0.05% Formic acid) at a flow rate of 0.4 mL/min. The initial condition of the gradient was 100% solution A; after 3.5 min the mobile phase was brought to 100% solution B and kept there for 1.5 min. Then the column was re-equilibrated to the initial condition for 1 min (total run time 6 min). The autosampler was kept at 10 °C, the injection volume was 10 µL. Data acquisition, data processing and system control were managed by MassLynx software (Waters, Milan, Italy). The mass spectrometer coupled to the UPLC system was set in positive ionisation mode (ESI+) with a capillary voltage of 3.5 kV, a source temperature of 150 °C and a desolvation temperature of 400 °C. The flow rate of the Nitrogen was 800 L/h for the desolvation and the cone flow rate was 60 L/h.

Ion monitoring was performed in multiple reaction mode, with the mass transitions and collision energies (CE) as reported here: CBD 315.14 → 193.04, CE 25; CBD-d3 318.10 → 196.14, CE 25; THC 315.11 → 193.05, CE 25; THC-d3 318.19 → 196.12, CE 25; CBDA 359.15 → 219.07, CE 30; THCA 359.13 → 219.11, CEC 30; CBN 311.15 → 223.10, CE 20 [23,24,25].

All the standard cannabinoid solutions necessary to create the calibration curve were diluted to concentrations between 1250 ng/mL and 5 ng/mL. CBD-d3 and THC-d3 were used as internal standards. 

All the samples to be analysed were diluted with isopropanol to obtain a final concentration suitable for the range of the calibration curve.

In order to analyse the oils dispensed in the capsules, the capsules were opened by separating the two halves. Both parts were then immersed in a sufficient quantity of isopropanol (10 mL) so that the oil from the capsules could mix with the solvent. The solid residue was thereafter separated from the liquid by centrifugation. It was subsequently diluted and analysed as described above.

Each time the stability of the oils was tested, the analysis was carried out in triplicate. As for the capsules, each test was performed on 10 capsules of the same lot.

### 4.5. Stability Test

The oils deriving from the β-4 procedure and the same oils contained in the capsules were tested at regular intervals (at least every 30 days) in order to evaluate the stability after refrigerated storage (2–8 °C) and storage at room temperature (15–25 °C). The analytical method employed is that described in Section 4.4.

### 4.6. Statistical Evaluation

For each of the active molecules of interest, the average content, the corresponding standard deviation as well as the maximum and minimum concentrations in a determined quantity of the finished product were evaluated.

## 5. Conclusions

The objective of the present work was to carry out a stability study for the oil extract obtained by a specific method developed by our research group. Furthermore, in order to facilitate the consumption of the prescribed medical *Cannabis* therapy by patients, a standard procedure was defined for the preparation of hard capsules containing the oil extract; thereafter, the quality and stability were evaluated. The encapsulation process did not alter the quantity of the active molecule present in the oil and the stability tests yielded excellent results.

The capsule form, thanks to its features (ease of transportation, neutral organoleptic properties and stability at room temperature for extended periods) would facilitate and ensure adherence to therapy on the part of the patients in treatment.

As widely discussed in literature, oral administration has a lower bioavailability (5–20%) than inhalation. Pharmacological effects range from 30 min to 3 h and the maximum concentration of cannabinoids in the blood is usually reached within 2 h. Despite this, oral administration is generally preferred, as it is easy to administer [26,27,28,29]. The use of the formulation developed during our studies, could therefore represent a promising option for the use in therapy.

Given that gastric juices may alter some of the components of the oil, such as CBD [26,27,28,29,30], further studies will address the issue of how to make the capsules containing oils prepared by the β-4 procedure gastro-resistant. Subsequently, an assessment will be performed of whether this type of formulation improves the bioavailability of the cannabinoids of interest.

## 6. Patent

An Italian patent was granted by the Italian Office for Patents and Brands for the procedure for *Cannabis* oil production (patent number 102018000011128, 17 November 2020).

## Figures and Tables

**Table 1:** Comparison oil–capsules.

	Oil	Capsules Containing 156 µL of Oil, T0					Capsules Containing 312 µL of Oil, T0				
Active Constituent	Average Concentration of Active Constituent (mg/mL) in Oil	Average Concentration of Active Constituent (mg/mL) in Capsules	Standard Deviation	Δ% Compared to Oil	Concentration of Active Molecule: Minimum Value (mg/mL) in Capsules	Concentration of Active Molecule: Maximum Value (mg/mL) in Capsules	Average Concentration of Active Constituent (mg/mL) in Capsules	Standard Deviation	Δ% Compared to Oil	Concentration of Active Molecule: Minimum Value (mg/mL) in Capsules	Concentration of Active Molecule: Maximum Value (mg/mL) in Capsules
CBD	12.092	12.057	0.42	−0.29	11.483	12.524	12.557	0.57	3.85	11.400	13.139
THC	6.971	7.216	0.21	3.51	6.873	7.455	7.286	0.46	4.51	6.348	7.661
CBDA	0.631	0.611	0.03	−3.25	0.580	0.657	0.630	0.02	0.11	0.608	0.676
THCA	0.036	0.037	0.00	1.667	0.034	0.039	0.036	0.00	−0.32	0.033	0.039
CBN	0.450	0.441	0.03	−2.078	0.412	0.489	0.455	0.02	1.16	0.429	0.494

T0: initial conditions.

**Table 2:** Oils stability.

						Oils-Fridge														
		OILS, T0				T15					T30					T60				
	Active Constituent	Average Concentration of Active Constituent (mg/mL)	Standard Deviation	Concentration of Active Molecule: Minimum Value (mg/mL)	Concentration of Active Molecule: Maximum Value (mg/mL)	Average Concentration of Active Constituent (mg/mL)	Δ% Compared to T0	Standard Deviation	Concentration of Active Molecule: Minimum Value (mg/mL)	Concentration of Active Molecule: Maximum Value (mg/mL)	Average Concentration of Active Constituent (mg/mL)	Δ% Compared to T0	Standard Deviation	Concentration of Active Molecule: Minimum Value (mg/mL)	Concentration of Active Molecule: Maximum Value (mg/mL)	Average Concentration of Active Constituent (mg/mL)	Δ% Compared to T0	Standard Deviation	Concentration of Active Molecule: Minimum Value (mg/mL)	Concentration of Active Molecule: Maximum Value (mg/mL)
	CBD	12.092	1.31	10.442	14.814	12.338	2.04	1.26	10.524	14.007	12.591	4.12	1.01	10.941	14.496	12.917	6.82	0.90	11.551	14.092
	THC	6.971	0.61	5.935	8.188	6.790	−2.60	0.43	6.309	7.452	6.781	−2.73	0.28	6.111	7.035	6.754	−3.11	0.15	6.510	7.039
	CBDA	0.631	0.07	0.504	0.783	0.573	−9.29	0.12	0.406	0.757	0.576	−8.81	0.10	0.423	0.706	0.608	−3.64	0.07	0.492	0.688
	THCA	0.036	0.09	0.010	0.070	0.034	−7.25	0.01	0.010	0.043	0.039	8.17	0.01	0.030	0.052	0.035	−4.59	0.02	0.017	0.064
	CBN	0.450	0.02	0.329	0.602	0.414	−7.92	0.04	0.357	0.494	0.412	−8.28	0.04	0.349	0.479	0.471	4.78	0.05	0.407	0.552
	**Oils-Fridge**																			
	**T90**					**T120**					**T150**					**T180**				
**Active Constituent**	**Average Concentration of Active Constituent (mg/mL)**	**Δ% Compared to T0**	**Standard Deviation**	**Concentration of Active Molecule: Minimum Value (mg/mL)**	**Concentration of Active Molecule: Maximum Value (mg/mL)**	**Average Concentration of Active Constituent (mg/mL)**	**Δ% Compared to T0**	**Standard Deviation**	**Concentration of Active Molecule: Minimum Value (mg/mL)**	**Concentration of Active Molecule: Maximum Value (mg/mL)**	**Average Concentration of Active Constituent (mg/mL)**	**Δ% Compared to T0**	**Standard Deviation**	**Concentration of Active Molecule: Minimum Value (mg/mL)**	**Concentration of Active Molecule: Maximum Value (mg/mL)**	**Average Concentration of Active Constituent (mg/mL)**	**Δ% Compared to T0**	**Standard Deviation**	**Concentration of Active Molecule: Minimum Value (mg/mL)**	**Concentration of Active Molecule: Maximum Value (mg/mL)**
CBD	10.994	−9.09	0.75	9.909	12.170	11.658	−3.59	1.25	10.136	13.632	11.566	−4.36	0.95	10.245	12.664	11.533	−4.63	0.68	10.700	12.826
THC	6.468	−7.22	0.36	5.897	6.943	7.134	2.34	0.51	6.204	7.715	7.108	1.96	0.26	6.773	7.566	6.790	−2.61	0.29	6.209	7.156
CBDA	0.658	4.28	0.10	0.535	0.846	0.682	8.08	0.12	0.514	0.886	0.657	4.14	0.11	0.501	0.817	0.688	8.94	0.08	0.588	0.844
THCA	0.038	3.90	0.02	0.012	0.056	0.033	−8.26	0.02	0.013	0.064	0.034	−6.73	0.02	0.010	0.050	0.039	7.34	0.02	0.010	0.061
CBN	0.417	−7.23	0.04	0.361	0.485	0.475	5.60	0.08	0.354	0.579	0.490	9.01	0.07	0.347	0.568	0.465	3.37	0.04	0.425	0.529
						**Oils-Room Temperature**														
		**Oils, T0**				**T15**					**T30**					**T60**				
	**Active Constituent**	**Average Concentration of Active Constituent (mg/mL)**	**Standard Deviation**	**Concentration of Active Molecule: Minimum Value (mg/mL)**	**Concentration of Active Molecule: Maximum Value (mg/mL)**	**Average Concentration of Active Constituent (mg/mL)**	**Δ% Compared to T0**	**Standard Deviation**	**Concentration of Active Molecule: Minimum Value (mg/mL)**	**Concentration of Active Molecule: Maximum Value (mg/mL)**	**Average Concentration of Active Constituent (mg/mL)**	**Δ% Compared to T0**	**Standard Deviation**	**Concentration of Active Molecule: Minimum Value (mg/mL)**	**Concentration of Active Molecule: Maximum Value (mg/mL)**	**Average Concentration of Active Constituent (mg/mL)**	**Δ% Compared to T0**	**Standard Deviation**	**Concentration of Active Molecule: Minimum Value (mg/mL)**	**Concentration of Active Molecule: Maximum Value (mg/mL)**
	CBD	12.092	1.31	10.442	14.814	11.725	−3.04	1.26	9.891	13.897	12.516	3.51	0.73	11.542	13.883	12.904	6.72	1.26	10.848	14.961
	THC	6.971	0.61	5.935	8.188	6.482	−7.02	0.45	5.646	7.240	6.633	−4.85	0.33	6.116	7.128	6.587	−5.52	0.19	6.905	6.402
	CBDA	0.631	0.07	0.504	0.783	0.570	−9.64	0.05	0.468	0.631	0.573	−9.22	0.04	0.503	0.629	0.598	−5.30	0.06	0.504	0.699
	THCA	0.036	0.09	0.010	0.070	0.036	−0.92	0.01	0.013	0.050	0.039	7.95	0.01	0.015	0.054	0.035	−2.75	0.01	0.017	0.056
	CBN	0.450	0.02	0.329	0.602	0.406	−9.60	0.03	0.362	0.451	0.435	−3.23	0.03	0.390	0.484	0.489	8.69	0.04	0.412	0.543
	**Oils-Room Temperature**																			
	**T90**					**T120**					**T150**					**T180**				
**Active Constituent**	**Average Concentration of Active Constituent (mg/mL)**	**Δ% Compared to T0**	**Standard Deviation**	**Concentration of Active Molecule: Minimum Value (mg/mL)**	**Concentration of Active Molecule: Maximum Value (mg/mL)**	**Average Concentration of Active Constituent (mg/mL)**	**Δ% Compared to T0**	**Standard Deviation**	**Concentration of Active Molecule: Minimum Value (mg/mL)**	**Concentration of Active Molecule: Maximum Value (mg/mL)**	**Average Concentration of Active Constituent (mg/mL)**	**Δ% Compared to T0**	**Standard Deviation**	**Concentration of Active Molecule: Minimum Value (mg/mL)**	**Concentration of Active Molecule: Maximum Value (mg/mL)**	**Average Concentration of Active Constituent (mg/mL)**	**Δ% Compared to T0**	**Standard Deviation**	**Concentration of Active Molecule: Minimum Value (mg/mL)**	**Concentration of Active Molecule: Maximum Value (mg/mL)**
CBD	11.101	−8.20	1.23	10.005	13.253	12.005	−0.72	1.07	10.302	13.340	12.123	0.26	0.77	10.687	13.018	11.921	−1.41	1.04	10.388	13.269
THC	6.426	−7.82	0.63	5.474	7.005	6.788	−2.63	0.37	6.319	7.341	6.942	−0.42	0.39	6.286	7.346	6.826	−2.09	0.16	6.584	7.084
CBDA	0.603	−4.44	0.08	0.397	0.709	0.667	5.65	0.07	0.578	0.804	0.627	−0.72	0.08	0.544	0.750	0.665	5.28	0.08	0.529	0.791
THCA	0.039	7.68	0.02	0.011	0.066	0.035	−3.67	0.02	0.014	0.062	0.039	7.92	0.02	0.010	0.061	0.034	−5.51	0.02	0.010	0.067
CBN	0.471	4.73	0.09	0.604	0.297	0.465	3.40	0.11	0.304	0.589	0.476	5.87	0.04	0.407	0.535	0.481	6.96	0.07	0.375	0.596

T0: initial conditions; T 15: 15 days; T 30: 30 days; T 60: 60 days; T 90: 90 days; T 120: 120 days; T 150: 150 days; T 180: 180 days.

**Table 3:** Capsule stability.

	Capsules Containing 156 µL of Oil											
	T90 Fridge						T180 Fridge					
Active Constituent	Average Concentration of Active Constituent (mg/mL) in Capsules	Standard Deviation	Δ% Compared to Oil	Δ% Compared to Cps at T0	Concentration of Active Molecule: Minimum Value (mg/mL) in Capsules	Concentration of Active Molecule: Maximum Value (mg/mL) in Capsules	Average Concentration of Active Constituent (mg/mL) in Capsules	Standard Deviation	Δ% Compared to Oil	Δ% Compared to Cps at T0	Concentration of Active Molecule: Minimum Value (mg/mL) in Capsules	Concentration of Active Molecule: Maximum Value (mg/mL) in Capsules
CBD	12.369	0.34	2.29	2.59	12.030	12.843	12.511	0.55	3.47	3.77	11.485	13.545
THC	7.351	0.24	5.45	1.87	7.029	7.603	7.420	0.21	6.44	2.83	7.135	7.640
CBDA	0.624	0.04	−1.11	2.27	0.570	0.680	0.637	0.03	0.95	4.32	0.592	0.676
THCA	0.036	0.00	0.00	-2.19	0.034	0.039	0.036	0.00	0.00	−1.64	0.033	0.039
CBN	0.458	0.01	1.78	3.98	0.442	0.471	0.455	0.03	1.11	3.25	0.414	0.491
	**T90 Room Temperature**						**T180 Room Temperature**					
**Active Constituent**	**Average Concentration of Active Constituent (mg/mL) in Capsules**	**Standard Deviation**	**Δ% Compared to Oil**	**Δ% Compared to Cps at T0**	**Concentration of Active Molecule: Minimum Value (mg/mL) in Capsules**	**Concentration of Active Molecule: Maximum Value (mg/mL) in Capsules**	**Average Concentration of Active Constituent (Mg/Ml) in Capsules**	**Standard Deviation**	**Δ% Compared to Oil**	**Δ% Compared to Cps at T0**	**Concentration of Active Molecule: Minimum Value (mg/mL) in Capsules**	**Concentration of Active Molecule: Maximum Value (mg/mL) in Capsules**
CBD	12.904	0.31	6.72	7.02	12.571	13.301	12.648	0.50	4.60	4.90	11.857	13.278
THC	6.923	0.37	−0.69	−4.06	6.286	7.255	7.349	0.17	5.42	1.84	7.111	7.592
CBDA	0.643	0.02	1.90	5.24	0.611	0.658	0.634	0.02	0.48	3.89	0.590	0.656
THCA	0.038	0.00	5.56	2.94	0.036	0.039	0.036	0.00	0.00	−3.01	0.033	0.039
CBN	0.441	0.04	−2.00	0.12	0.486	0.488	0.444	0.03	−1.33	0.73	0.406	0.481
	**Capsules Containing 312 µL of Oil**											
	**T90 Fridge**						**T180 Fridge**					
**Active Constituent**	**Average Concentration of Active Constituent (mg/mL) in Capsules**	**Standard Deviation**	**Δ% Compared to Oil**	**Δ% Compared to Cps at T0**	**Concentration of Active Molecule: Minimum Value (mg/mL) in Capsules**	**Concentration of Active Molecule: Maximum Value (mg/mL) in Capsules**	**Average Concentration of Active Constituent (mg/mL) in Capsules**	**Standard Deviation**	**Δ% Compared to Oil**	**Δ% Compared to Cps at T0**	**Concentration of Active Molecule: Minimum Value (mg/mL) in Capsules**	**Concentration of Active Molecule: Maximum Value (mg/mL) in Capsules**
CBD	12.471	0.82	3.13	−0.69	10.977	13.283	12.325	0.61	1.93	−1.85	10.946	12.874
THC	6.767	0.35	−2.93	−8.21	6.441	7.345	7.076	0.37	1.51	−4.02	6.475	7.595
CBDA	0.652	0.03	3.33	3.36	0.62	0.691	0.649	0.03	2.85	2.96	0.601	0.692
THCA	0.037	0.00	2.78	2.44	0.035	0.039	0.037	0.00	2.78	1.30	0.033	0.039
CBN	0.455	0.03	1.11	−1.16	0.406	0.494	0.444	0.02	−1.33	−3.42	0.411	0.485
	**T90 Room Temperature**						**T180 Room Temperature**					
**Active Constituent**	**Average Concentration of Active Constituent (mg/mL) in Capsules**	**Standard Deviation**	**Δ% Compared to Oil**	**Δ% Compared to Cps at T0**	**Concentration of Active Molecule: Minimum Value (mg/mL) in Capsules**	**Concentration of Active Molecule: Maximum Value (mg/mL) in Capsules**	**Average Concentration of Active Constituent (mg/mL) in Capsules**	**Standard Deviation**	**Δ% Compared to oil**	**Δ% Compared to Cps at T0**	**Concentration of Active Molecule: Minimum Value (mg/mL) in Capsules**	**Concentration of Active Molecule: Maximum Value (mg/mL) in Capsules**
CBD	12.618	0.51	4.35	0.48	11.886	13.279	12.52	0.50	3.54	−0.30	11.833	12.988
THC	6.701	0.47	−3.87	−9.10	6.317	7.595	7.066	0.17	1.36	−4.15	6.624	7.618
CBDA	0.647	0.03	2.54	2.67	0.604	0.686	0.629	0.02	−0.32	−0.14	0.598	0.684
THCA	0.035	0.00	−2.78	−3.62	0.033	0.037	0.037	0.00	2.78	1.90	0.034	0.039
CBN	0.449	0.03	−0.22	−1.379	0.41	0.495	0.453	0.03	0.67	−1.51	0.422	0.487

T0: initial conditions; T 90: 90 days; T 180: 180 days.

## Data Availability

Data is contained within the article.

## Abbreviations

THCA	delta-9-tetrahydrocannabinolic acid
CBDA	cannabidiolic acid
THC	delta-9-tetrahydrocannabinol
CBD	cannabidiol
CBN	cannabinol
CE	collision energies
